# The Influence of Flame Exposure and Solid Particle Erosion on Tensile Strength of CFRP Substrate with Manufactured Protective Coating

**DOI:** 10.3390/ma17051203

**Published:** 2024-03-05

**Authors:** Przemysław Golewski, Michał Budka

**Affiliations:** 1 Department of Solid Mechanics, Faculty of Civil Engineering and Architecture, Lublin University of Technology, Nadbystrzycka 40 Str., 20-618 Lublin, Poland; 2 Laboratory of Civil Engineering, Faculty of Civil Engineering and Architecture, Lublin University of Technology, Nadbystrzycka 40 Str., 20-618 Lublin, Poland; m.budka@pollub.pl

**Keywords:** solid particle erosion, flame exposure, carbon fibre-reinforced polymer (CFRP), protective layer, tensile test

## Abstract

This paper presents the results of laboratory tests for new materials made of a carbon fibre-reinforced polymer (CFRP) composite with a single-sided protective coating. The protective coatings were made of five different powders—Al_2_O_3_, aluminium, quartz sand, crystalline silica and copper—laminated in a single process during curing of the prepreg substrate with an epoxy matrix. The specimens were subjected to flame exposure and solid particle erosion tests, followed by uniaxial tensile tests. A digital image correlation (DIC) system was used to observe the damage location and deformation of the specimens. All coatings subjected to solid particle erosion allowed an increase in tensile failure force ranging from 5% to 31% compared to reference specimens made of purely CFRP. When exposed to flame, only three of the five materials tested, Al_2_O_3_, aluminium, quartz sand, could be used to protect the surface, which allowed an increase in tensile failure force of 5.6%.

## 1. Introduction

PMC (polymer matrix composite) materials have been used since the mid-1930s, when Owens Corning was the first company in the world to start an industry in this field using glass fibres as reinforcement [1]. Since then, composites have become widespread in many technical fields, such as aerospace, the automotive industry, shipbuilding, wind turbines and civil engineering. Despite their many advantages, such as favourable weight-to-strength ratio, composites also have disadvantages, such as, among others, the ease with which they can be damaged, e.g., by erosion or high temperatures, and the difficulty of repair and recycling. Therefore, the entire design and manufacturing process must take place with great care to avoid failures early on in the life of the product. Despite this, some application ranges remain unattainable or very limited. It becomes necessary to apply suitable coatings on their surface to protect or provide functional properties. In terms of coatings on the surface of PMC composites, various solutions can be found in the literature, e.g., the use of:

- Flame retardant resin additives [2,3];
- Nanopaper with carbon nanofibres [4];
- Intumescent or ceramic mats [5,6,7];
- Flame spray or cold spray [8,9];
- Sol–gel technique [10];
- Powders mixed with resin [11].

Another example would be unwanted coatings that form naturally as oxides and need to be removed. Ref. [12] proposes the use of a numerical method to calculate the effects of particles through blasting on a rust-covered surface, and points out that the material parameters of the tested coatings are crucial in the model simulation process.

Coatings applied to the surface of PMC composites can serve different functions. Polyurea as an outer layer can be used as protection against low-velocity impact or point static loading [13,14,15]. Energy absorption during impact can be increased by as much as 94% compared to pure CFRP. Structures such as aircraft fuselages or wind turbine blades are exposed to lightning strikes [16]. In this case, the authors in [17] used polyaniline to produce the coating. It was found that with a 345 μm-thick PANI-based LSP system, 83% and 100% of the original strength of the sample can be achieved for composites such as CFRP and GFRP, respectively, loaded with a discharge of 40 kA to 100 kA. Electrical conductivity of the coating can also be achieved by chemical application of nickel [18] or by using a copper grid [19]. A difficult issue is the protection of the composite substrate against high temperatures arising, for example, as a result of a fire or when part of the structure has to operate at elevated temperatures. The authors in [20] used gypsum-based coatings. These are relatively thick (25 mm) and have an irregular surface, but allow the CFRP composite to be protected in a fire for 2 h, which meets the British standards for building materials. An order of magnitude less thick are the coatings proposed in [21] that also serve as a barrier against high temperatures. In this case, a poly(vinyl alcohol) (PVA)-based coating containing ammonium polyphosphate (APP) and sepiolite nanofillers (SP) was used. A glass fabric interlayer was still required between this material and the laminate. This allowed the temperature of the flame-retardant surface of the sample to be lowered. There is no information on the mechanical strength of the coatings in the two articles mentioned above. In terms of mechanical loading, both the gypsum-based and PVA-based material may not have sufficient strength for particle erosion or impact. Hence, other methods are sought, e.g., based on different types of powders mixed with resin and applied to the sample surface. In Ref. [22], a new thermal barrier was proposed using Al_2_O_3_ particles. The fabrication of the samples was carried out in two stages. First, a thermal barrier was produced by combining Al_2_O_3_ powder with a ceramic binder, while in the second stage, RTM (resin transfer moulding) technology was used to produce a composite with the previously prepared barrier. The resin partially fills the porous space of the thermal barrier and a mechanical bond between the two materials is created. Bending tests carried out for specimens loaded with a 700 °C flame prove that 50% of the substrate strength can be retained. The authors in [23] proposed an original technology for producing the protective layer using a special powder that is a mixture of diglycidyl ether of bisphenol A (DGEBA), polyester resins, mineral fillers and pigment. The whole procedure is carried out in several steps and involves firstly curing the layer, and only then is the prepreg layer laid down and re-cured. Hence, the authors in [24] proposed a new, less complex technology, which has the advantage of making the coating in one process while the prepreg is cured. This type of coating can be formed from different metal powders or oxides, while the formed coating has very good adhesion to the CFRP substrate. Table 1 summarizes the latest developments in coating manufacturing on PMC composites.

To date, there are no results in the literature on the effects of flame or solid particle erosion for a coating formed such as that presented in [24]. In the previous article, the authors used the same technology as now, but the specimens were not subjected to any thermal or erosive effects and were tested in three-point bending tests using acoustic emission, while the objective was to determine whether sudden and early delamination of the coatings would occur. The results of the tests showed that delamination does not occur, even with large deformations of the substrate; hence, in the present work, samples with manufactured coatings of powders—Al_2_O_3_, aluminium, quartz sand, crystalline silica and copper—were subjected to flame exposure and solid particle erosion tests followed by uniaxial tensile tests. In the case of specimens subjected to solid particle erosion, all of the proposed materials fulfilled their function and allowed an increase in tensile failure force in the range of 5% to 31%, while for flame exposure, only the first three of the above-mentioned can be used for which this increase was at the level of 5.6%.

## 2. Materials and Methods

**Figure 1:**
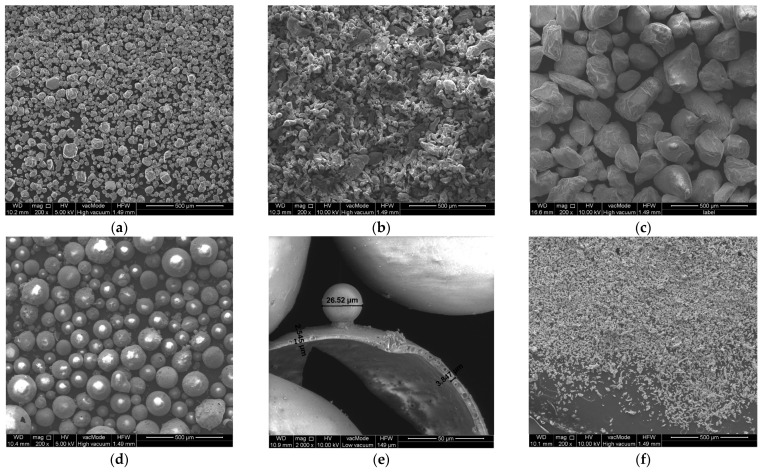
SEM images: (**a**) Al_2_O_3_, (**b**) aluminium, (**c**) quartz sand, (**d**) crystalline silica (×200), (**e**) crystalline silica (×2000) (**f**) copper.

A total of five chemically and physically different powders—Al_2_O_3_, aluminium, quartz sand, crystalline silica and copper (PolyCore, Świdnik, Poland)—were selected to produce the coatings. The properties of the materials are summarised in Table 2, while images of the powder grains taken using SEM are shown in Figure 1 for the same ×200 magnification. The first material (Al_2_O_3_) is widely used in technology and commercially available for different powder gradations. It is relatively inexpensive and is used for the manufacture of grinding wheels or blasting. It has a high compressive strength and Young’s modulus, as well as a much lower thermal conductivity compared to metals, and hence it can be a candidate for both erosion and high-temperature coatings. The second material, aluminium, has the advantage that it can deform plastically, which will not result in brittle cracking under high mechanical loads. In addition, compared to low-carbon steel, it does not corrode as quickly atmospherically and is almost a third of the weight. Quartz sand is the cheapest and most accessible material. Like Al_2_O_3_, it has high strength and low thermal conductivity. Quartz sand has been used on a large scale in façade composite panels on a building of the Museum of Modern Art in San Francisco [29], serving both a decorative and protective function. The fourth material is a lightweight filler in the form of crystalline silica. The grains are spherical and the inside is hollow, as shown in Figure 1e. The wall thickness is approximately 3–4 μm. Hence, a coating made of this material will have the advantage that the weight of the final product will not increase significantly. The last material is copper. It is the most expensive considering all the materials presented, so its use must be well thought out and justified. The copper-coated samples shown in Appendix A are highly aesthetic and attractive. Hence, such a product can find use in the construction industry, e.g., replacing copper in sheets for roofing. Copper has another advantage, antimicrobial properties [30], which can be used as an advantage in the construction of facade panels, which are often covered by fungi and moulds [31].

All samples were made using Kordcarbon epoxy prepreg (Fiberpreg GmbH, Neu-Ulm, Germany) by Wit-Composites. The technology for making the samples comprised the following steps:

1. Preparation of the flat mould, cutting and layering of the prepreg;
2. Laying of a 1 mm thick tool to form the powder layer on the prepreg;
3. Manual moulding of the powder within the limits of the tool;
4. Removing the tool and preparing the vacuum pack;
5. Forming of samples in the autoclave according to the prepreg manufacturer’s instructions.

**Figure 2:**
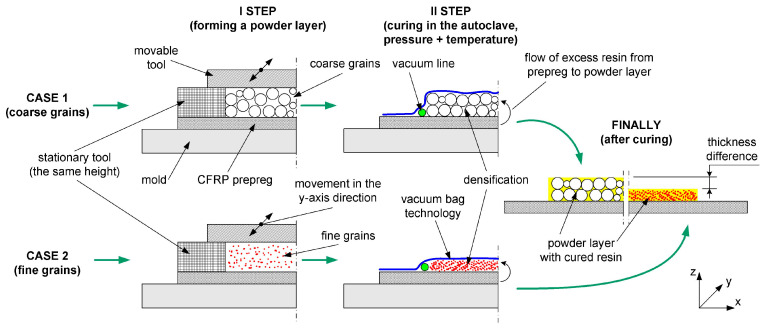
Process of making the protective coating.

**Figure A1:**
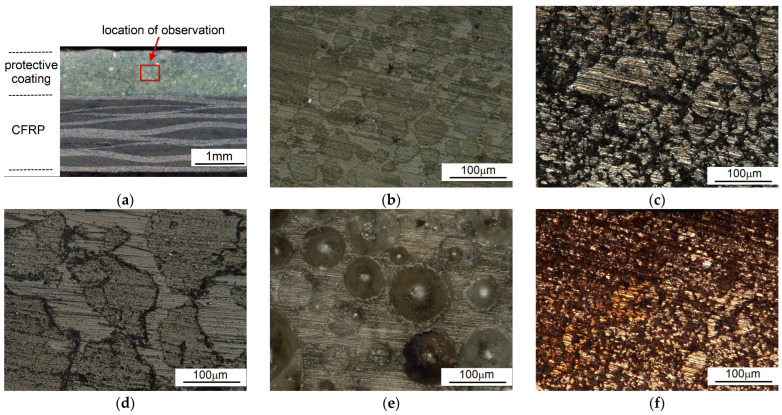
Microstructure of coatings: (**a**) cross-sectional view of the sample, (**b**) Al_2_O_3_, (**c**) aluminium, (**d**) quartz sand, (**e**) crystalline silica, (**f**) copper.

The photographs of the workflow are analogous to those presented in [24]. The whole process is also explained in Figure 2. In each case, the thickness of the stationary layer-forming tool was the same. However, densification occurred during the process—less for coarse grains and more for fine powder. This resulted in different thicknesses (Table 3). The microstructure of the manufactured coatings can be seen in the Appendix A in Figure A1.

After curing, the specimens were cut with a CNC plotter to a size of 30 mm × 250 mm. Then, the samples were subjected to thickness measurements using a digital micrometre with a range of 0–25 mm with output to a computer made by Mitutoyo, whose readings in millimetres were to three decimal places.

The samples were divided into two main groups:

T—specimens exposed to flame.

E—samples exposed to solid particle erosion.

There were 5 batches of samples with protective coatings in each group. There were 5 samples in each batch. In addition, there were reference samples (3 each) in both groups that did not have a protective coating. Hence, the total number of samples was 56.

**Figure 3:**
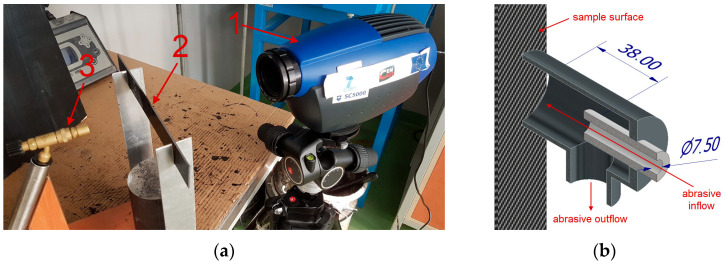
Schematics of laboratory stands: (**a**) for flame exposure (1—thermal imaging camera, 2—sample, 3—gas burner); (**b**) for solid particle erosion test.

Group T specimens were exposed to the flame using the test stand shown in Figure 3a. This type of stand is often used for comparative tests, e.g., [33,34], where a gas torch and digital temperature measurement are used. The distance of the burner from the surface of the sample was set using a K-type thermocouple so that the temperature in front of the sample was in the range 750–800 °C. This is a temperature that can occur, for example, during a developed fire in a building or in hot sections of turbine engines. For this purpose, an ORCA 3 burner (Metalurgica Orca Ltd., Sao Paulo, Brazil) and isobutane gas (Alpen Camping, Schilpario, Austria) were used. A FLIR SC5000 thermal imaging camera was used to record the temperature distribution on the opposite surface of the sample. The flame exposure time in each case was 30 s. The results from the thermal imaging camera were processed in the Altair software (version 5.91.010.).

Group E specimens were subjected to a point solid particle erosion, as shown in Figure 3b, using an abrasive blast gun (Tagred TA 1358 (Nowe, Poland)). The angle between the eroding jet and the sample surface was 90° analogous to [35]. The erosion was carried out at one point of the specimen in its axis. The exposure time of the blast was 3 s, the installation pressure was 0.6 MPa, the inner diameter of the nozzle was 7.5 mm and the distance of the nozzle from the sample surface was 38 mm. Electrocorundum with a gradation of F60 was used as the abrasive.

After flame and particle erosion tests, the specimens were prepared for uniaxial tensile tests by bonding 30 mm × 50 mm tabs to the ends. The tabs were made from 1 mm thick PFCC 201 laminate (Izo-Erg, Gliwice, Poland) and bonded with Epidian 5 epoxy resin with PAC hardener (CIECH Sarzyna S.A., Nowa Sarzyna, Poland).

Microscopic observations were made using a Keyence VHX-7000 (Osaka, Japan) digital microscope. Uniaxial tensile tests were performed using an MTS 100 kN testing machine (Eden Prairie, MO, USA) and with an Aramis digital image correlation system (Lenso Ltd., Poznań, Poland). The speed at which the specimens were loaded was 1 mm/min. The results from the testing machine were processed in Diadem 2019.

## 3. Results

In the presented work, the results are divided into two subsections on the effects of flame and erosion. In the first case, the results are more extensive, as a thermal imaging camera was used to record the temperature distribution and further process these results. In the case of particle erosion, the results relate only to mechanical tensile tests.

The results of the work carried out are so extensive that photographs of the flame exposure tests and the solid particle erosion tests (the stage before the strength tests) are included in Appendix A.

### 3.1. Flame Exposure Test Results

**Figure 4:**
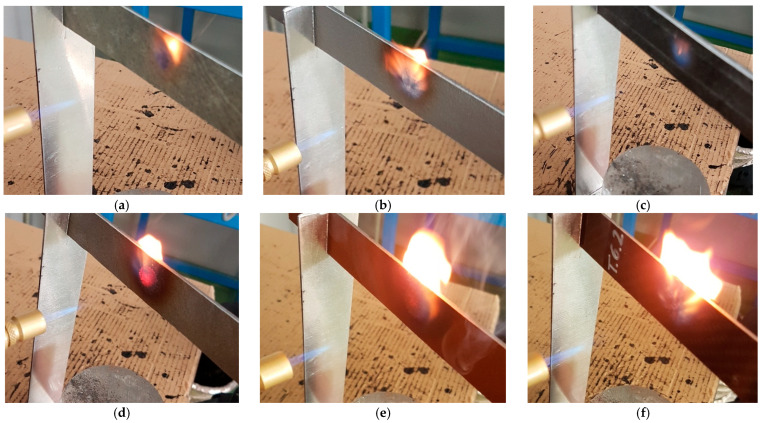
Performing flame exposure tests: (**a**) Al_2_O_3_, (**b**) aluminium, (**c**) quartz sand, (**d**) crystalline silica, (**e**) copper, (**f**) reference.

Flame exposure tests for each material were carried out for 30 s. The time was chosen so that damage to the coating was visible, but there was no complete degradation of the substrate. Figure 4 shows the flame effects for each material. In almost every batch, the surface of the sample was ignited after several seconds. An exception is the coating made of quartz sand. This is the material with the largest grains of those tested. Crystalline silica, which is a lightweight filler, behaves very unfavourably; it is in the form of spheres that are hollow inside. When these are damaged, a porous structure with a large heat transfer surface area is formed (Figure 1e), which is easily heated.

**Figure 5:**
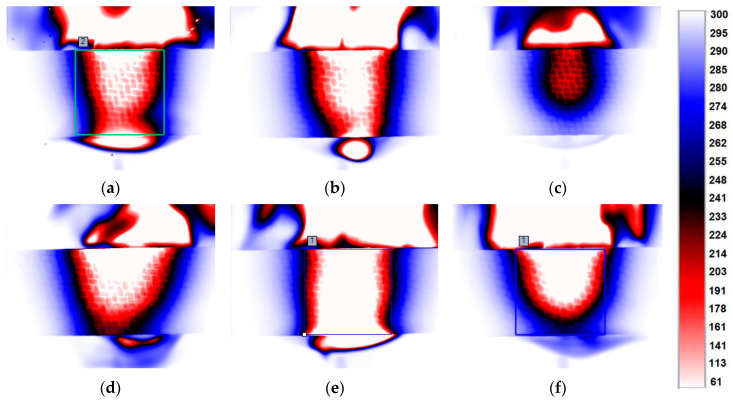
Thermal camera images for 30 s flame exposure: (**a**) Al_2_O_3_, (**b**) aluminium, (**c**) quartz sand, (**d**) crystalline silica, (**e**) copper, (**f**) reference.

Throughout the flame exposure, the temperature on the back of the sample was recorded. Due to the extensive nature of the results, Figure 5 shows images of the temperature distribution after 30 s for each of the materials tested. For each sample, a 30 mm × 30 mm area was defined in Altair software, for which the minimum, maximum and average temperatures could be determined. Both temperature distribution maps and values are extremely important from a practical point of view as well as for numerical modelling.

**Figure 6:**
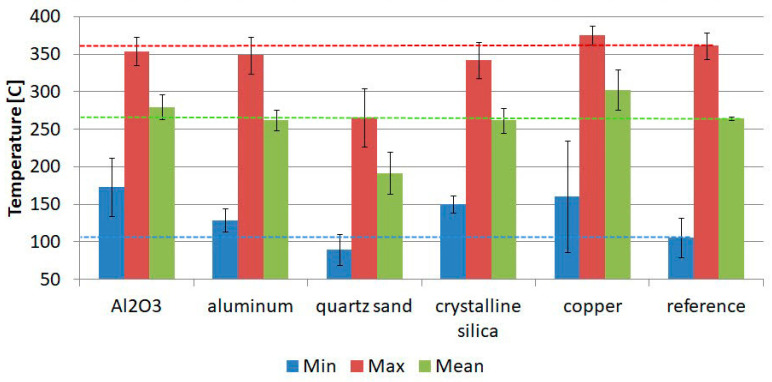
Batch-averaged temperature values on a 30 mm × 30 mm field for 30 s flame exposure.

A summary of the results for 30 s of heating for a 30 mm × 30 mm field is presented in Figure 6. These are the averaged results for each batch. The standard deviation bars are included. To simplify the analysis, horizontal lines were also drawn in Figure 5 to refer to the batch of reference samples. The most favourable material for building a protective coating is quartz sand. It has a thermal conductivity coefficient of 1.5 W/m·K. The decrease in the average temperature value compared to the reference samples was approximately 27.5%. The worst-performing material is copper. Copper is a good thermal conductor with a heat conduction coefficient of 260 W/m·K, and thus there was an increase in the mean surface temperature of approximately 14.5% compared to the reference samples. Furthermore, it should be noted that, as shown in Table 3, uniform coating thicknesses were not achieved. A low thermal conductivity of 46 W/m·K is also possessed by Al_2_O_3_, but with this grain size and coating thickness, the advantages of this material did not become apparent. Similar results to Al_2_O_3_ were also obtained for aluminium and crystalline silica-coated samples.

**Figure 7:**
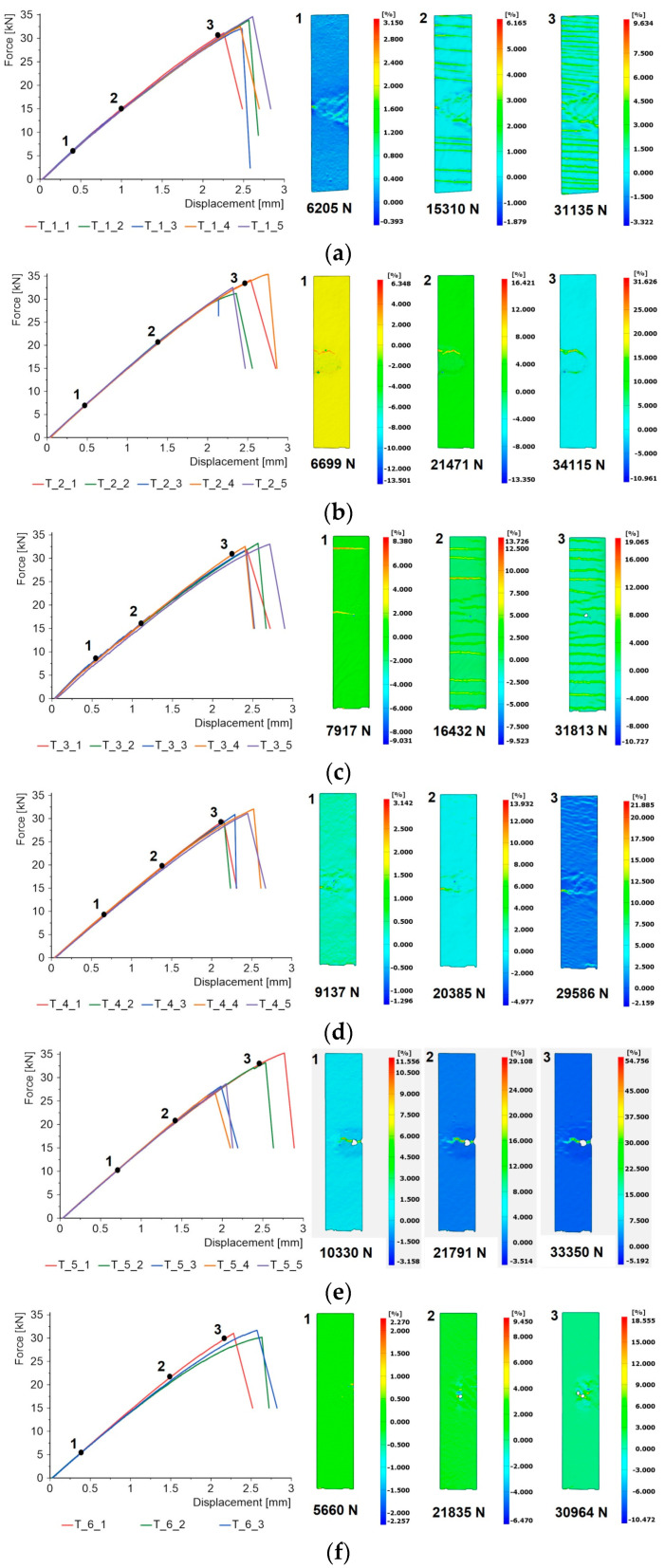
Force–displacement diagrams and principal strain maps for specimens subjected to flame exposure: (**a**) Al_2_O_3_, (**b**) aluminium, (**c**) quartz sand, (**d**) crystalline silica, (**e**) copper, (**f**) reference.

Figure 7 presents the results from uniaxial tensile tests on specimens subjected to flame exposure. Throughout the tensile process, the displacements of the specimen surface were recorded using the DIC system. After processing, these were presented in the form of principal strain maps. For each specimen, three images were selected for different loading levels: at an early stage, at half load and at the final stage before the specimen failed. In each case, the strain maps refer to the first sample in the batch.

The force–displacement diagrams are all linear, with the failure of the specimen occurring in a sudden manner. However, the strain maps revealed that coating damage can occur much earlier, even before the maximum force is reached. This is particularly evident for two materials, Al_2_O_3_ and quartz sand. For these materials, the fields are not homogeneous and horizontal lines appear at an early stage of tensile strain, indicating that cracks are occurring in the coating.

However, these cracks do not grow and further ones appear in the sample. The reason for the formation of cracks is that both oxide materials do not deform plastically. In the case of copper and aluminium, the strain fields are uniform outside the flame area. Similarly for the reference sample, homogeneous strain fields are also obtained. The visible white blank fields in the copper sample and the reference sample in the flame-affected zone are due to the fact that the camera system has lost its reference points, probably as a result of paint spalling due to significant deformation and poor adhesion.

### 3.2. Solid Particle Erosion Test Results

**Figure 8:**
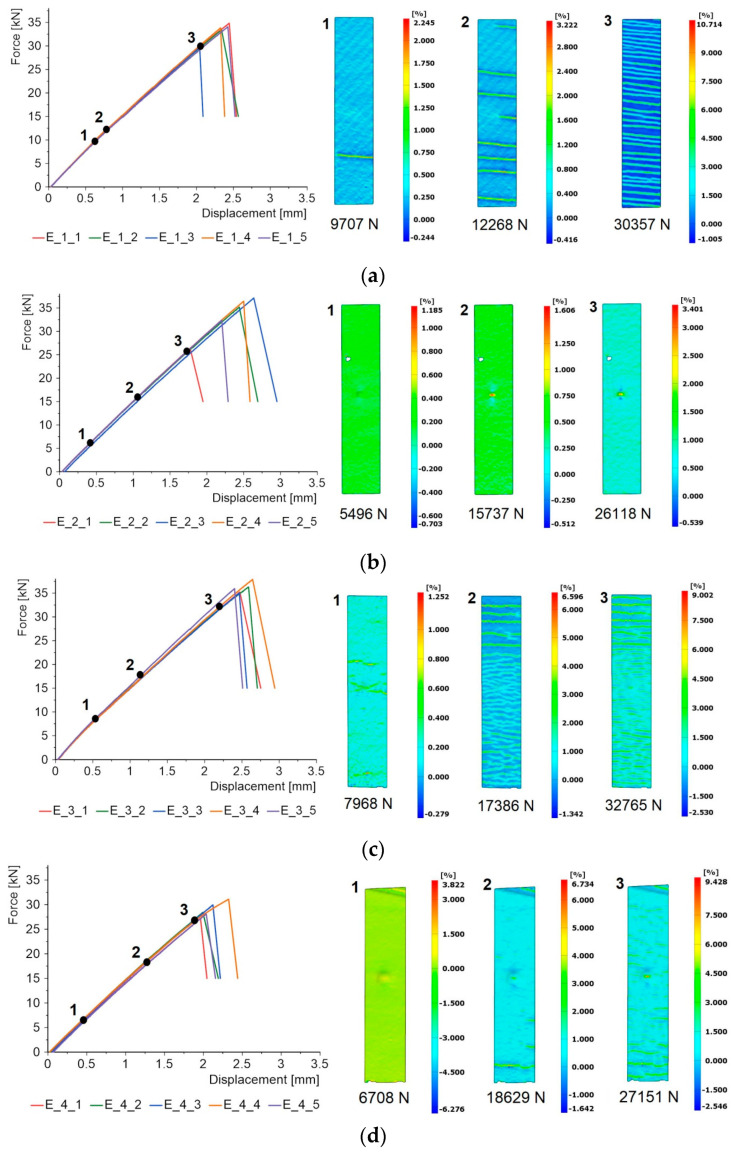

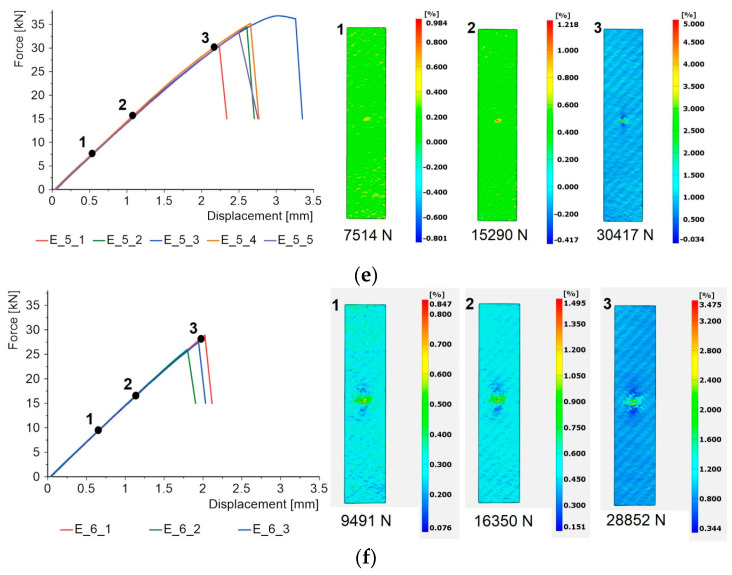
Force–displacement diagrams and principal strain maps for specimens subjected to erosion exposure: (**a**) Al_2_O_3_, (**b**) aluminium, (**c**) quartz sand, (**d**) crystalline silica, (**e**) copper, (**f**) reference.

The results for the specimens subjected to solid particle erosion tests are shown in Figure 8.

As with the specimens subjected to flame exposure tests, the results for the principal strain fields for the three load levels are also presented. The graphs are also linear and there is a sudden failure of the specimen. For specimens with a coating made of oxides (Al_2_O_3_ and quartz sand), horizontal lines representing brittle cracking in the coating also appear. However, the intensity of these cracks is more pronounced than for the flame-exposed samples. The reason for this may be that the effect of the high temperature was not limited to the flame area, but the sample was heated along its entire length. Hence, the mechanical properties of the epoxy resin matrix may be reduced, its stiffness decreases, and thus the intensity of the strain maps also decreases.

Considering the strain maps for the coatings made of aluminium and copper, these are homogeneous. However, throughout the tensile stage, the point in the specimen axis where the intensity of erosion was greatest is visible.

## 4. Discussion

The thickness of the achieved coatings should be considered the first issue in the discussion. The average thickness of the substrate was 2.075 mm and the bulk thickness of the powder layers was 1 mm. This means that theoretically, the thickness of the whole sample should be around 3 mm. However, as Table 3 shows, this dimension is not achievable. This is due to the fact that the samples were autoclaved under pressure and the grains fill the voids between each other and further indent into the substrate, which becomes elastic during curing. Such a phenomenon can be considered positive for two reasons. Firstly, as a result of the reduction of the voids between the grains, there is no significant outflow of resin from the prepreg. Secondly, such a layer becomes more compact and stronger than if there are loose grains with a large amount of resin, which could occur during moulding with, for example, the non-pressurised manual mixing of powder and resin.

However, the disadvantage of such a technology is the difficulty in designing a suitable layer thickness, because each time the powder material, grain size and planned thickness are changed, a technological test must be carried out in advance. This is thus a topic for future research.

In the following analysis, it is important to take a closer look at applied technology. On the one hand, it is innovative and has advantages such as:

- The production of the coating in one process, during the curing of the prepreg;
- Very good adhesion to the CFRP substrate, as presented in [24];
- The possibility of using different powders and mixtures of powders;
- High aesthetic qualities of the outer surface.

On the other hand, it should be noted that this technology is imperfect due to the manual formation of the layer. This may even become impossible with small thicknesses of the order of 0.1 mm. Therefore, the use of mechanical feeders and numerically controlled powder distribution on the surface should be considered.

**Figure 9:**
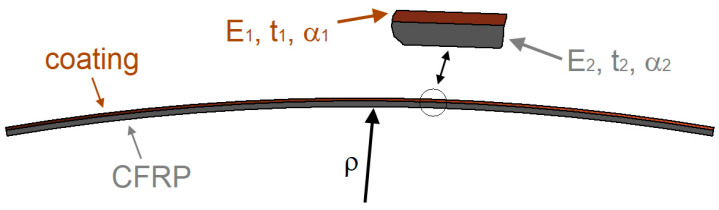
Deformation of the sample after removal from the autoclave.

Another disadvantage of the presented solution is that as a result of the difference in thermal expansion of the substrate material and the forming coating, the sample will be slightly bent when cooled and removed from the autoclave (Figure 9).

The deformation will depend on both the coating material used and the thickness ratio of the substrate to the coating and can be determined analytically using Equation (1) [36].

(1) $$\rho =\frac{t\left[3\cdot {\left(1+m\right)}^{2}+\left(1+m\cdot n\right)\left(m^{2}\cdot \frac{1}{m\cdot n}\right)\right]}{6\cdot \left(\alpha_{1}-\alpha_{2}\right)\left(T_{h}-T_{c}\right){\left(1+m\right)}^{2}}$$

where:

$m=\frac{t_{1}}{t_{2}}$—ratio of coating thickness to composite thickness,

$n=\frac{E_{1}}{E_{2}}$—ratio of Young’s modulus of the coating to the composite,

$t=t_{1}+t_{2}$—sum of coating and substrate thickness,

α_1_, α_2_—coefficients of thermal expansion for the coating and the composite,

T_h_, T_c_—sample curing temperature and ambient temperature.

Equation (1) applies to isotropic materials. In this case, it would be necessary, using a representative volume element, to determine the Young’s modulus and coefficient of expansion for CFRP and the protective coating. On the other hand, literature data [37,38] and Table 2 can be used to make calculations for the quartz sand-coated sample for which the smallest radius of curvature was obtained. If the following data are provided—t_1_ = 1.2 mm, t_2_ = 2.07 mm, E_1_ = 74 GPa, E_2_ = 40 GPa, α_1_ = 16.41 × 10^−6^ 1/°C, α_2_ = 10 × 10^−7^ 1/°C, T_h_ = 125 °C, T_c_ = 22 °C—then the radius of curvature will be 1.128 m.

In the considered specimens, the radii of curvature and the curvature for each specimen are summarised in Table 3. These are results based on sample measurements using a digital sensor. If we compare the result from Table 4 for the quartz sand coating with the calculation from Equation (1), the difference is 18.7%.

Specimens with a quartz sand coating have the highest curvature, while those with a copper coating have the lowest. Therefore, when designing products, especially flat products, using this technology, numerical simulations should be carried out in advance to verify the deformation of the composite product. The occurrence of curvature also leads to the appearance of additional residual stresses in the structure. However, in order to prevent this, it is possible, for example, to form the coating on both sides.

At present, almost all research papers that can be found in the literature focus on forming the coating on a flat surface. An exception is [39], in which both the original technology and a curved moulder were used. The use of flat specimens, on the one hand, greatly simplifies the whole technological and research process, but on the other hand, it should be kept in mind that composite parts such as wind turbine blades or boat hulls have a curved shape. Hence, the authors of this article are currently working on a technology that will allow coatings to be formed easily and efficiently also on curved surfaces.

**Figure 10:**
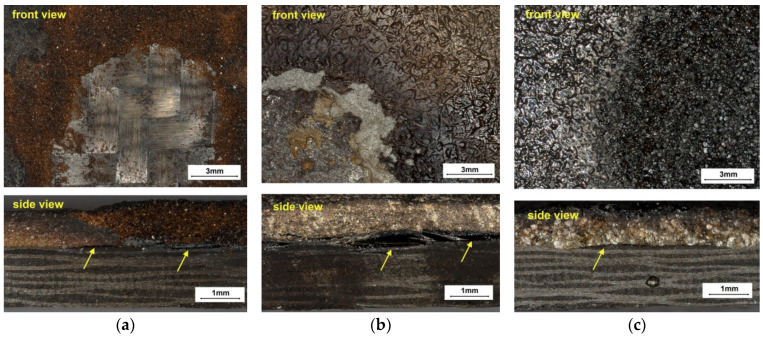

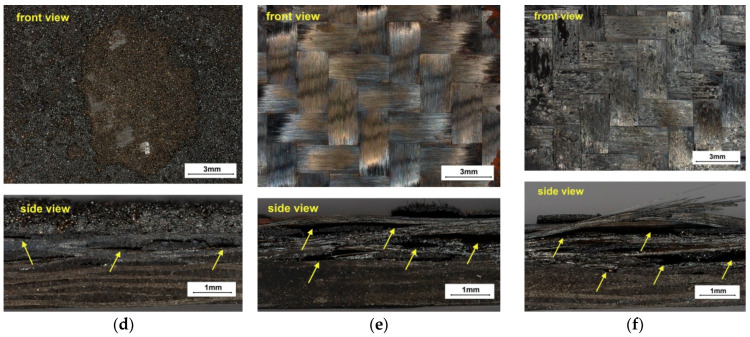
Surface view of specimens for flame exposure tests: (**a**) Al_2_O_3_, (**b**) aluminium, (**c**) quartz sand, (**d**) crystalline silica, (**e**) copper, (**f**) reference.

**Figure A2:**
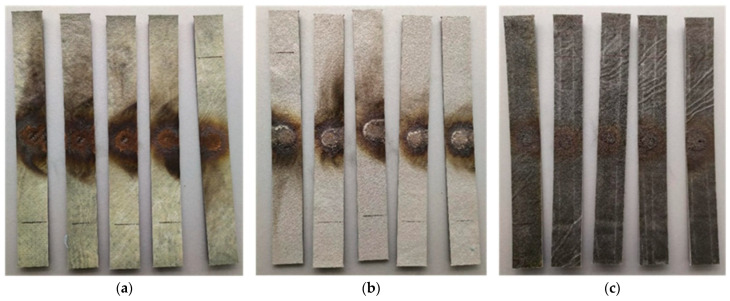

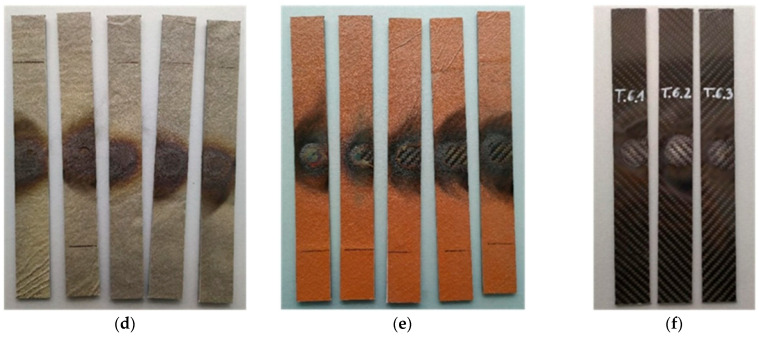
View of samples after flame exposure tests: (**a**) Al_2_O_3_, (**b**) aluminium, (**c**) quartz sand, (**d**) crystalline silica, (**e**) copper, (**f**) reference.

Turning to the results of the flame exposure tests, it should be noted that extremely different results were obtained with the five coatings. After the flame exposure tests, the samples were cleaned of soot and loose particles with a steel brush. A view of the surface from the flame exposure side and the side of the specimen is presented in Figure 10. A view of the whole specimens is also available in Appendix A in Figure A2.

Flame effects can be divided into two main groups: when there is no uncovered substrate and when the substrate is uncovered. The first group will include only two materials: aluminium and quartz sand. The second group, in order of the most uncovered substrate, will include copper, Al_2_O_3_ and crystalline silica. However, no matter which layer is used, a barrier effect still arises if we compare the effects with Figure 10f for a pure CFRP substrate. The absence of any barrier results in significant degradation and deformation in the PMC composite. Important information is shown by the images in Figure 10 on the side of the sample. For the coating made of quartz sand, no negative changes in the substrate are visible. Only a slight delamination of the coating occurs. This type of delamination without damage to the substrate itself is also visible for the Al_2_O_3_ coating, but in this case, as mentioned above, there is an uncovering of a significant area of the substrate. In the aluminium coating, evidence of thermal damage to the substrate are already visible, but these do not extend as deeply as for the copper or crystalline silica coating.

**Figure A3:**
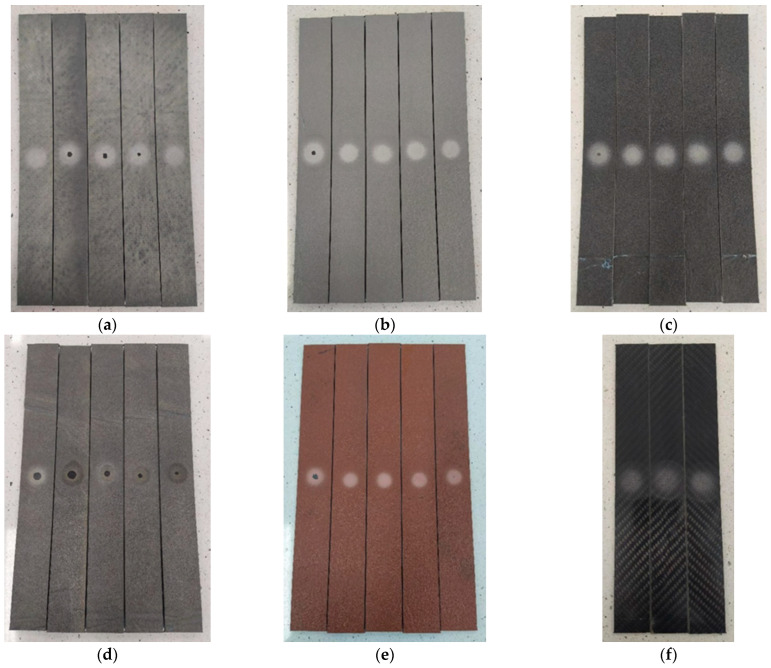
View of samples after erosion tests: (**a**) Al_2_O_3_, (**b**) aluminium, (**c**) quartz sand, (**d**) crystalline silica, (**e**) copper, (**f**) reference.

The samples subjected to particle erosion can be seen in Appendix A in Figure A3. In this case, two effects can also be distinguished: uncovering of the composite substrate and no uncovering. In fact, in each batch, there was a minimum of one sample in which the composite surface was uncovered to a greater or lesser extent. Such an effect could be due to a locally smaller coating thickness or a different intensity of stream exposure, e.g., a pressure surge in the compressed air system caused by switching on the compressor. However, there are undoubtedly two materials whose application is not favourable: crystalline silica and Al_2_O_3_. For the first material, the substrate was uncovered in every sample, and for the second in three samples. The most surprising effect is for the copper coating, as it was the thinnest. For copper, aluminium and quartz sand, substrate uncovering was observed for only one sample in the series.

**Figure 11:**
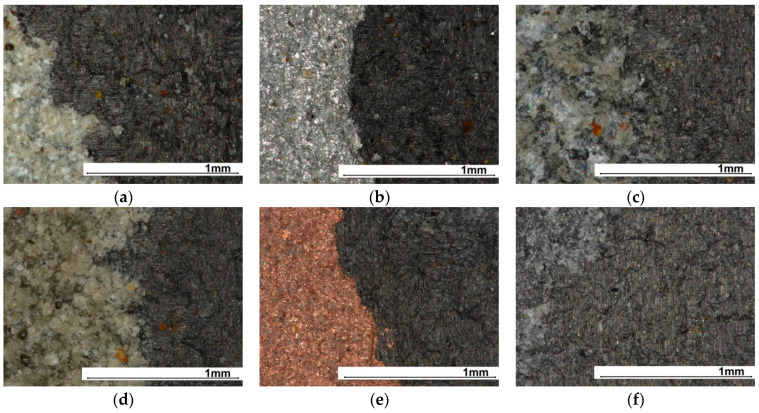
Surface view of specimens for solid particle erosion: (**a**) Al_2_O_3_, (**b**) aluminium, (**c**) quartz sand, (**d**) crystalline silica, (**e**) copper, (**f**) reference.

Figure 11 summarises the results of the microscopic observations for the samples in which substrate uncovering occurred. The images show the boundary zone between the coating and the substrate. The erosion is so intense that not even a single grain remains on the CFRP surface.

**Figure 12:**
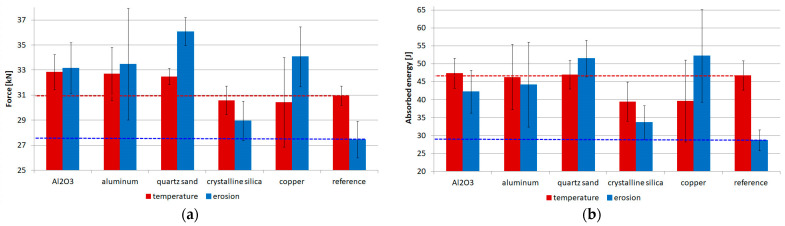
Results of uniaxial tensile tests: (**a**) summary of results averaged over a batch of maximum forces, (**b**) summary of results averaged over a batch of absorbed energy.

For the designer, who could possibly apply one of the selected coatings, it is important how the aggressive environment will affect the strength. The effectiveness of the respective coatings was verified in a uniaxial tensile test, and the results are collected in Figure 12a for maximum forces and Figure 12b for absorbed energy. In the present tests, for the substrate subjected to the exposure to flame and particle erosion, values of 30.96 kN and 27.46 kN of failure forces were obtained, respectively. These are the levels to which the results for the other samples will be related, and hence the horizontal lines in Figure 12a for easier analysis. The reason that the reference specimens obtained a higher force for the flame test than for the erosion test can be explained by the fact that the high temperature does not damage the fibres themselves, but the matrix, while the fibres are degraded by erosion.

Considering the effect of flame exposure, these results confirm previous microscopic observations. Materials such as crystalline silica and copper resulted in a reduction in maximum force of approximately 1.7%. For other materials, an increase of about 5.6% was obtained, which is a favourable phenomenon.

When considering the results for specimens subjected to solid particle erosion, in this case, each of the proposed coatings performs well and an increase in failure force ranging from 5% to 31% was obtained for crystalline silica and quartz sand respectively. This increase is due to the fact that part of the load is transferred through the coating, which is a great advantage in contrast to paints or intumescent mats, which are characterised by low stiffness and thus cannot take part in the load transfer. It is also worth noting that the smallest standard deviation was obtained for the quartz sand samples.

Figure 12b displays the results for the energy absorbed during the tensile tests, which corresponds to the area under the force–displacement diagram. In this case, similar conclusions can be drawn. Considering the flame exposure, the absorbed energy is at the same level for coatings made of Al_2_O_3_, aluminium and quartz sand, while it is about 15% lower for the coating made of crystalline silica and copper. In the case of erosion, an increase in absorbed energy ranging from 17% (crystalline silica) to 82% (copper) was obtained for all coatings.

## 5. Conclusions

This paper presents the application of a new technology to produce five different coatings on CFRP substrates and discusses its advantages and disadvantages in relation to current methods and future expectations. A total of 56 samples were made, which were divided into two groups and subjected to flame and solid particle erosion exposure. In the final stage, the specimens were subjected to uniaxial tensile tests. The conducted work allows the following conclusions to be drawn.

- The use of coating formation technology in a single process during the curing of the prepreg results in residual stresses that, depending on the ratio of substrate thickness to layer thickness, can lead to deformation of the product.
- With the quartz sand coating, a 27.5% reduction in average substrate temperature was achieved compared to the reference samples. For the copper-coated samples, the situation is reversed and the substrate temperature increases by 14.5%.
- The application of the DIC method allowed the surface strain of the samples to be observed. For coating materials such as Al_2_O_3_ and quartz sand, the appearance of horizontal cracks was observed, the number of which intensifies when the load is increased. Such cracks occur over the entire surface irrespective of the location of flame or erosion damage. Samples with coatings made of metal powders (aluminium and copper) are characterised by homogeneous strain fields outside the damage area.
- Considering flame exposure, an increase in failure force compared to the reference value was obtained for samples with coatings made of Al_2_O_3_, aluminium and quartz sand. The increments in failure force were at the level of 5.6%. This shows that the fabricated coatings can be considered barriers against the effects of high temperature.
- All of the proposed coatings can be used for erosion protection. Increases in tensile failure force ranging from 5% to 31% were obtained for crystalline silica and quartz sand, respectively.
- Of all the proposed coatings, considering both flame and erosion exposure, the quartz sand coating shows the best results. This is evidenced not only by the force increases compared to the reference samples and microscopic images but also by the smallest standard deviation, which was 0.65 kN and 1.13 kN for flame and erosion exposure, respectively.

## Figures and Tables

**Table 1:** Literature reports of coating manufacturing on composite substrates.

Type and Thickness of the Substrate	Type and Thickness of Coating Material	Method of Manufacturing/Joining the Coating	Type of Load/Test	References
CFRP 4.1 mm	Ti/TiN, 1.5 to 11 μm	PVD (physical vapour deposition)	sand erosion, rain erosion	[25]
CFRP 2.4–3.85 mm	Al_2_O_3_ powder and ceramic binder, 1.45 mm	VARTM (vacuum-assisted resin transfer moulding)	500–700 °C flame exposure, flexural strength	[22]
GFRP 3.7 mm	(PVA)-based coating 2.8 mm	vacuum infusion process	cone calorimeter tests 50 kW/m^2^	[21]
CFRP 2 mm and GFRP 2.4 mm	polyaniline coating, 249–427 μm	manual application, curing at 130 °C	lightning strike	[17]
CFRP 1.5 mm	copper, quartz sand, Al_2_O_3_, aluminium, crystalline silica, microballoon, 0.3–1 mm	curing process in the autoclave	3-point bending tests	[24]
CFRP 2 mm	polyurea, 0.5 mm–1 mm	spraying process, curing for 7 days	quasi-static indentation and low velocity impact	[13]
CFRP 1.5 mm	aluminium bond coat + top coat (four different ceramic materials coating, 0.8 mm)	plasma sprayed	mechanical properties—Young’s modulus	[26]
CFRP 1.7 mm	graphene nano platelet-based coating, prepreg 62 μm	compression moulding process (3 bar), cured for 1 h at 120 °C	laser heating with power density 25–150 kW/m^2^	[27]
CFRP 1.2–3 mm	non-intumescent fireproof coating, 15 mm, 25 mm, 35 mm	curing process for a duration of 20 days	large-space fire, standard fire, bending tests	[20]
GFRP 3.2 mm	CNF-based nanopaper, 0.3 mm	RTM (Resin Transfer Moulding)	cone calorimeter tests, post fire 3-point bending tests	[4]
GFRP 2.4 mm	flame-retarded epoxy resin and ceramic particles (Ce) 1.09 mm, (Zr) 0.89 mm, (Re) 0.94 mm	hand lay-up method	cone calorimeter tests: 20, 30, 40, 50 kW/m^2^	[28]

**Table 2:** Properties of materials used in the manufacture of coatings [32].

	Al_2_O_3_	Aluminium	Quartz Sand	Crystalline Silica	Copper
Density (kg/m^3^)	3690	2700	2650	500	8940
Young’s modulus (GPa)	370	70	74	-	110
Tensile strength (MPa)	300	240	155	-	365
Compressive strength (MPa)	3000	240	1600	-	365
Vickers hardness	1365	83	1100	-	90
Melting temperature (°C)	2050	630	1650	1200	1066
Thermal conductivity (25 °C) (W/mK)	46	200	1.5	-	260

**Table 3:** Sample thicknesses [mm].

	Al_2_O_3_	Aluminium	Quartz Sand	Crystalline Silica	Copper	References
Sample 1	2.41	2.40	3.46	2.48	2.24	2.05
Sample 2	2.41	2.54	3.53	2.75	2.25	2.13
Sample 3	2.54	2.43	3.40	2.60	2.33	2.05
Sample 4	2.46	2.54	3.05	2.72	2.16	
Sample 5	2.45	2.49	2.91	2.56	2.18	
Mean	2.45	2.48	3.27	2.62	2.23	2.07

**Table 4:** Results of curvature measurements of the samples.

	Al_2_O_3_	Aluminium	Quartz Sand	Crystalline Silica	Copper
radius “r” of curvature [m]	2.538	2.811	1.388	2.443	9.527
curvature 1/r [1/m]	0.39	0.36	0.72	0.41	0.1

## Data Availability

The data presented in this study are available on reasonable request from the corresponding author.
